# Oral Health and Care for Elderly People with Alzheimer’s Disease

**DOI:** 10.3390/ijerph17165713

**Published:** 2020-08-07

**Authors:** Sherry Shiqian Gao, Chun Hung Chu, Fanny Yuk Fun Young

**Affiliations:** 1 Faculty of Dentistry, The University of Hong Kong, Hong Kong 999077, China; chchu@hku.hk; 2 Department of Business Administration, Hong Kong Shue Yan University, Hong Kong 999077, China; drfyoung@gmail.com

**Keywords:** oral health, oral hygiene, Alzheimer’s disease, dementia

## Abstract

Dementia is one of the main causes of disability among elderly people. It is a progressive neurodegenerative disease that affects elderly people’s ability to perform daily living activities. Alzheimer’s disease is the main subtype of dementia and causes declining memory, reasoning, and communication skills. They also have behavioural and psychological symptoms, such as depression and aggression. It is essential for them to maintain good oral health, as oral health is an important and integral part of their general health. Neglecting oral health allows dental diseases to develop, and these diseases are difficult and costly to treat. However, dental diseases can be treated with ambulatory care rather than hospitalisation and emergency care. Elderly people should establish daily oral hygiene care routines during the early stages of Alzheimer’s disease. They should have regular dental examinations and early minimal interventions to prevent the need for extensive and complicated procedures. Maintaining oral health becomes challenging, however, when Alzheimer’s disease progresses to the middle and late stages. Because elderly people might forget or lose interest in keeping their teeth healthy, caretakers and community health workers may need to take over this task. Dentists should provide guidance on the maintenance of oral health, as the techniques used to provide this support vary depending on the elderly people concerned. The purpose of this paper is to provide an overview of oral health and the importance of oral care for elderly people with Alzheimer’s disease. The paper also discusses appropriate dental interventions and techniques for maintaining good oral health and helping people with Alzheimer’s to enjoy a satisfactory quality of life.

## 1. Introduction

Improvements in standards of living and people’s quality of life have contributed to an increase in life expectancy and thus an ageing population [1]. Increasing age is an important risk factor for Alzheimer’s disease. The World Health Organization defined Alzheimer’s disease as a neurodegenerative disease of unknown aetiology, characterised by progressive memory and cognitive impairment [2]. It is the most common form of dementia, accounting for around 50–80% of dementia cases worldwide [3]. In fact, the prevalence of Alzheimer’s disease worldwide has doubled during the past couple of decades [4].

Alzheimer’s disease is costly not only as a terminal disease but also financially and socially. On the financial side, as elderly people lose their ability to engage in work, they become financially dependent on their family members. In the latter stages, employing caregivers or being institutionalised may be necessary. On a larger scale, a number of government subsidies are spent on providing services and facilities for such elderly people. In 2015, the annual socioeconomic cost of Alzheimer’s disease in China was US $168 billion. In addition, it was estimated to increase to US $507 billion in 2030 and US $1890 billion in 2050 [5]. In other words, the alarming financial burden of care is becoming heavier rather quickly. In addition, taking care of elderly people with Alzheimer’s disease can be exhausting. Family members may need to take time off from work, which can impact their livelihoods. As the behavioural issues of these elderly individuals worsen, family members and caretakers may suffer emotionally [6].

Unfortunately, Alzheimer’s disease has no known cause [7]. The age of onset for this degenerative disorder is usually 65 or above [6]. Elderly people often present with cerebral cortical atrophy and show a significant increase in intracellular neurofibrillary tangles due to tau protein hyperphosphorylation as well as neurotic plaques containing beta-amyloid [8]. Elderly people may suffer from the progressive loss of intellectual and social abilities that will eventually interfere with daily functioning [9]. Diagnosis is usually made based on the history of signs and symptoms [7]. Elderly individuals may live for four to eight years after diagnosis [10].

The clinical onset of Alzheimer’s disease can be divided into three stages: preclinical, mild cognitive impairment, and Alzheimer’s disease dementia [11]. The preclinical stage of Alzheimer’s disease pathology begins many years before the onset of Alzheimer’s disease symptoms [12]. The preclinical stage includes three periods. In the first and second periods, certain cerebrospinal fluid biomarkers become detectable [13]. However, patients still do not display any dementia symptoms. In the third period of the preclinical stage, the patients may perform worse on cognitive function tests compared with the previous periods [14]. The second stage of Alzheimer’s disease is mild cognitive impairment. Patients’ cognitive ability can decline in various domains, including memory, language, attention and executive function [15]. The third stage is Alzheimer’s disease dementia. When the elderly people have at least two of the following symptoms: impaired reasoning, impaired memory for new information, impaired language function, impaired visuospatial ability, or changes in behaviours or personality, they will be diagnosed with dementia [16]. Sadly, Alzheimer’s disease is progressive and incurable [17]. In addition, elderly people with Alzheimer’s disease may present with dental conditions compounded with impaired self-care, frailty, polypharmacy, co-existing morbidity, malnutrition, xerostomia, dysphasia, dysphagia, the risk of aspiration pneumonia, and impaired cognition, which undermines informed consent [6,9,17,18].

## 2. Oral Health of Elderly People with Alzheimer’s Disease

The oral health of elderly people with Alzheimer’s disease can be worse than that of those without dementia [19]. A recent systematic review found that elderly people with dementia have higher incidents of coronal and root caries, retained roots and plausible causes of orofacial pain [20]. Moreover, elderly people with dementia generally have oral hygiene issues, which are to be expected due not only to age but also to dementia [8]. Specifically, they tend to have more prevalent periodontal problems, including gingival bleeding, periodontitis, and attachment loss. They may also suffer from xerostomia and oral lesions, such as stomatitis and Candidiasis.

In Alzheimer’s disease, reduced cognition and dexterity certainly complicate oral hygiene measures, so help with oral hygiene practices may be necessary [21]. Inferior oral hygiene definitely contributes to periodontal inflammation [22]. Meanwhile, xerostomia may stem from ageing, prescribed medication or any co-morbidity, such as post-radiation therapy [3]. In addition, elderly people with Alzheimer’s disease can have low stimulated and unstimulated submandibular salivary flow rates [22]. In the case of hyposalivation and/or differences in the buffering capacity of saliva, xerostomia may accentuate plaque accumulation, periodontal inflammation halitosis, the caries rate, Candidiasis and an uncomfortable denture fit, thus creating a vicious cycle of oral health that will inevitably impact the elderly individual’s quality of life [23].

Interest has grown in the association among tooth loss, periodontal disease and dementia [21,24]. Tooth loss is a common indicator of poor oral health [20]. The association between tooth loss and dementia could be bidirectional in nature [25]. With tooth loss, masticatory function is adversely affected. The impaired masticatory manoeuver will affect nutritional intake and may lead to reduced cerebral stimulation and blood flow; thus, favouring the development and/or worsening of dementia [25]. A systematic review by Pazos et al. [21] showed that a definite association between periodontal disease and dementia is still inconclusive. However, a plausible mechanism of periodontal disease that is conducive to the development of Alzheimer’s disease involves pathogens from the oral cavity entering the bloodstream and crossing the blood-brain barrier more readily as the person ages. This causes inflammation and increases the formation of senile plaques, which are conducive to Alzheimer’s disease [19,21]. Another postulated mechanism concerns the inflammatory hypothesis of Alzheimer’s disease. Chronic periodontitis results in an increased level of inflammatory products in the bloodstream and thus may cause cerebral inflammation. The chronic inflammation operates similarly to the vicious cycle that leads to neurodegeneration [26]. Finally, a bidirectional association could exist between periodontal disease and Alzheimer’s disease with both conditions potentiating each other [21].

## 3. Importance of Oral Care for Elderly People with Alzheimer’s Disease

Aspiration pneumonia is the misdirection of oropharyngeal or gastric contents into the pulmonary parenchyma [27]. It is a common type of lung infection and may cause elderly death [9]. Risk factors for aspiration pneumonia in elderly people include age and frailty, dysphagia, diminished cough reflex, immunosuppression, medication (such as sedatives and anti-depressants), impaired cognition, decreased salivary clearance, xerostomia, and inadequate oral hygiene [27,28]. However, the oral hygiene of people with Alzheimer’s disease is usually unsatisfactory [22]. Therefore, it is crucial to improve the oral hygiene statuses of elderly people with Alzheimer’s disease to lower their risk of suffering from aspiration pneumonia.

Malnutrition is another issue associated with poor oral health. A lot of elderly people with Alzheimer’s disease suffer from poor oral health [22]. Compromised oral health (e.g., tooth loss, xerostomia) in elderly people impairs the sensory and masticatory functions; thus, adversely affecting their nutritional intake [19].

A longitudinal study found significantly more caries and restorations in elderly people with dementia compared with those without dementia [29]. Advanced age (older than 80 years), having more teeth and having a previous high caries experience were positive risk factors for an increase in caries. In other words, given the clinical course of Alzheimer’s disease, elderly people’s oral conditions are liable to worsen with age, thus potentially generating vicious cycles for systemic and local complications. Given their foreseeable inferior and deteriorating oral conditions, the presence of oral facial pain seems likely. Yet, the number of self-reports on pain has been fewer than expected [20]. It is unclear whether the nature of Alzheimer’s disease causes a reduced perception of pain [8], or if short-term memory loss and/or communication issues in elderly people with dementia can explain the situation [30]. The concern is when pain—The body’s way of signalling a problem—Is present but goes undetected, thus causing elderly people to suffer.

Xerostomia is a dry-mouth symptom that can be drug induced or may be a subjective or objective phenomenon of physiological aging [31]. Elderly people with Alzheimer’s disease may be on multiple medications that cause xerostomia [10]. A systematic review reported that elderly people with Alzheimer’s disease presented reduced stimulated and resting submandibular salivary flows [8]. The ill effects of xerostomia on oral health can include dental caries, periodontal disease, fungal infections, denture stomatitis, and masticatory discomfort [32]. In addition, xerostomia potentiates the adverse effects that poor oral health has on the quality of life of elderly people. For instance, pain from oral diseases, burning mouth syndrome, reduced food intake, impaired salivary cleansing, taste, mastication, and swallowing may invariably influence the systemic and psychological well-being of elderly people; thus, impacting their daily living activities and reducing their joy and comfort [8]. All of these add to the importance of oral care for elderly people.

Self-care is a definitive issue with elderly people with Alzheimer’s disease, especially as the disease progresses [19]. Poor oral hygiene invariably predisposes elderly people to poor oral health [22]. However, elderly people with Alzheimer’s disease may have behavioural and co-operative issues when confronted with the idea of receiving necessary dental services [33]. In addition, limited mobility, advanced age, poor systemic conditions, and frailty may limit their treatment options [6]. Furthermore, impaired short-term memory and cognition definitely highlight the importance of informed consent and communication with caretakers [17]. All of these may call for special arrangements, such as an in-hospital setting, at times. Given such considerations, focusing on oral care to prevent oral disease and to reduce the need for complicated dental treatment is advantageous [17]. This is feasible when caregivers can provide good oral healthcare to elderly people with Alzheimer’s disease [29].

## 4. Appropriate Interventions for Maintaining Good Oral Health

The appropriate dental intervention depends on the patient’s needs, preferences and concept of good oral health. At the end of the day, for elderly people with Alzheimer’s disease, important considerations will invariably include the longevity of the treatment outcome, the ease of maintenance, the maintenance of the masticatory function, and the prevention/deferral of inevitable dental disease [30]. Other considerations include access to care, co-existing medical conditions, and financial capability.

Professional ethics dictates respect and considerations for all patients. Elderly people with Alzheimer’s disease are entitled to just as much. Irrespective to the severity of the disease, we had better show our respect for the elderly people [6]. Our attitude will be apparent, not just to elderly people, but also to the caretaker and family members with whom we earnestly need to work. Moreover, the elderly people may not complain even if dental issues are bothering them [30]. Thus, friendly and effective communication and rapport with both elderly people and caretakers are essential during the taking of medical histories and during examinations. In this regard, we must pay attention to both verbal and non-verbal communication, such as being gentle and reassuring in speech, as well as making appropriate eye contact so that the elderly people feel comfortable and reassured [30]. Elderly people often have other medical conditions, such as hypertension and diabetes, and may be on multiple medications. Appointments may be designed to be short for elderly people’s low tolerance as well. Furthermore, the tell-show-do technique may facilitate treatment delivery. The use of mouth props and treating the patient in the semi-supine position for comfort and safety may also be necessary [10].

No doubt that with impaired memory and cognition in elderly people with Alzheimer’s disease, informed consent can be challenging. Therefore, a responsible person for informed consent must be established [10]. Informed consent is a process that respects elderly people’s needs and preferences for the most appropriate treatment. It not only requires a full understanding of the information but also ensures patient autonomy without coercion [34]. Naturally, involving the caretaker or family members in the informed consent process is not just advantageous but also necessary. Given the natural course of Alzheimer’s disease, elderly people may become increasingly dependent on the caretakers and family members. The caretakers and family members often have to assist the elderly people in oral hygiene, communication and even the informed consent process [35]. Consequently, building rapport, educating, and liaising with the caretakers is an important part of dental intervention [9].

## 5. Treatment Planning

The goals of treatment planning for older adults are to control dental disease, re-establish and preserve function, and enhance the quality of life [36]. The following factors likely influence service delivery: costs and affordability, needs, and demands (objective and subjective values and expectations, quality of life), accessibility to services (such as being homebound), maintenance and self-care (such as dexterity issues), medical condition and functional requirements (such as tube feeding) [30,36]. Hence, the dentist needs to strike a sensible balance when striving to achieve oral health and serving elderly people with Alzheimer’s disease [35]. To such ends, a few models are aimed at assisting in formulating the most appropriate management for individual cases. Examples of these models include the OSCAR Five-Point Geriatric Dental Assessment, the Seattle Care Pathway and the SOAP (Subjective findings, Objective findings, Assessment and Plan) [17,36]. The OSCAR Five-Point Geriatric Dental Assessment focuses on five key areas during the planning of oral health management for elderly people: oral (oral cavity), systemic (health history), capability (for self-care), autonomy (consent to care) and reality (financial, life expectancy, end-of-life care, etc.) [17]. The Seattle Care Pathway helps dentists to evaluate patients’ functional statuses and possible risks to their oral health, and then develop oral health plans [17]. The SOAP note is a method of documentation used to record patients’ information in a highly organised way [36]. All of these models are case sensitive; they can help practitioners to better understand patients’ statuses, needs, and risks. Although these models are not specifically introduced to elderly people with Alzheimer’s disease, the basic concepts can be considered when one is developing tailor-made dental care plans for elderly people with Alzheimer’s disease. The imminent risks of a decline in oral health should be considered during treatment planning for patients. Applying “caries management by risk assessment” is helpful for incorporating preventive measures, and “minimally invasive dentistry” in restorative work is conducive to a lasting functional outcome [17,35]. Due to the progressive nature of Alzheimer’s disease, developing a care (treatment) plan according to each stage of Alzheimer’s disease is desirable.

### 5.1. Early Stage of Alzheimer’s Disease

The aim of dental intervention should be prevention oriented, with long-lasting results and easy maintenance, as well as attending to the elderly’s needs (appearance, mastication) and circumstances (self-care and environmental issue) [10]. For example, controlling the disease may be best accomplished with relatively long-lasting and simple restorations that elderly people can easily maintain and that the dentist can easily remedy as the Alzheimer’s disease progresses [6,10]. In addition, preventative programs should be instilled early on when elderly people are still astute and competent, as this is important for both preventing dental diseases and maintaining self-care [10,30]. Fluoride therapy is an important strategy for caries prevention. Maintaining oral health is also possible with more frequent dental examinations and professional oral hygiene care, including scaling intervals [9]. In terms of self-care, patient education (oral hygiene instructions, dietary advice) must be tailored to address individual circumstances (dexterity and memory issues). Novelty devices may include an electric toothbrush, a water flosser, or a Collis Curve toothbrush [6,37]. In addition, innovative strategies can be used to mark dentures, supervise the oral hygiene routine or use the 5S methodology to develop an oral hygiene routine [3,9,10,37]. In addition, educating caretakers can help with maintaining oral hygiene [9].

### 5.2. Moderate Stage of Alzheimer’s Disease

Resistant behaviour is common during the moderate stage of Alzheimer’s disease [10]. Managerial considerations may include mouth props for safety, treating in the supine position to reduce the risk of aspiration, and having short appointments [10]. Apart from prevention, the aim of this stage of treatment is to maintain the dental status with simple treatments [6,10]. Provisional treatments should be considered to avoid complicated treatments. The atraumatic restorative technique and sedation may also be considered for appropriate cases.

### 5.3. Late Stage of Alzheimer’s Disease

By the late stage of Alzheimer’s disease, elderly people may become unmanageable [10]. In this situation, the aim of treatment would shift to be more palliative oriented and to provide emergency care. Sedation/general anaesthesia may be considered for appropriate cases [10]. In addition, an interdisciplinary approach is often essential for looking after the co-morbidities of elderly people. The education of the caretaker and family members is also essential.

## 6. Keeping a Satisfactory Quality of Life for Elderly People with Alzheimer’s Disease

The quality of life for elderly people often rests on their health, their bodies’ physical functioning and their psychosocial well-being. To various extents, the way in which oral health allows for satisfactory mastication and preferred aesthetics can contribute to elderly people’s general quality of life. Due to their cognitive impairment, it is difficult to deduce how much elderly people with Alzheimer’s disease appreciate their oral conditions [8]. Nonetheless, a healthy oral cavity will undeniably contribute to their quality of life. Satisfactory mastication efficiency; clean, aesthetic dentition; and a prosthesis could also contribute to quality of life [25] in terms of self-esteem and personal image. Perhaps the underlying contributory factor is to make nutritional intake possible, thus keeping the body healthy and functioning well [38]. To such ends, geriatric prosthodontic care with both fixed and removable prostheses as appropriate is justified for the appropriate cases [36]. In any case, oral hygiene measures and co-operation with caretakers and family members are definitely the keys to maintaining oral health.

## 7. Conclusions

Elderly people with Alzheimer’s disease usually have compromised oral hygiene due to their impaired memory and function. Developing and maintaining good oral health practices can help to improve their oral health and quality of life. Patients’ statuses, capabilities, and needs should be taken into account when one is making oral health care plans for elderly people with Alzheimer’s disease.
